# *Rhaphidophora korthalsii* modulates peripheral blood natural killer cell proliferation, cytokine secretion and cytotoxicity

**DOI:** 10.1186/1472-6882-13-145

**Published:** 2013-06-24

**Authors:** Swee Keong Yeap, Abdul Rahman Omar, Wan Yong Ho, Boon Kee Beh, Abdul Manaf Ali, Noorjahan Banu Alitheen

**Affiliations:** 1 Institute of Bioscience, University Putra Malaysia, Serdang, Selangor, Malaysia; 2 Department of Veterinary Pathology and Microbiology, Faculty of Veterinary Medicine, University Putra Malaysia, Serdang, Selangor, Malaysia; 3 Department of Cell and Molecular Biology, Faculty of Biotechnology and Biomolecular Sciences, University Putra Malaysia, 43400, Serdang, Selangor, Malaysia; 4 Faculty of Agriculture and Biotechnology, University Sultan Zainal Abidin, Kampus Kota, Jalan Sultan Mahmud, Kuala Terengganu 20400, Malaysia; 5 Department of Bioprocess Technology, Faculty of Biotechnology and Biomolecular Sciences, University Putra Malaysia, 43400, Serdang, Selangor, Malaysia

**Keywords:** *Rhaphidophora korthalsii*, NK cell, Immunomodulation

## Abstract

**Background:**

*Rhaphidophora korthalsii* (Araceae) is a root-climber plant which has been widely used in Chinese traditional medicine for cancer and skin disease treatment. Previous reports have recorded its immunomodulatory effects on mice splenocyte and human peripheral blood. This study investigated the potential immunostimulatory effect of *Rhaphidophora korthalsii* on human PBMC enriched NK cell.

**Methods:**

PBMC was exposed to various concentrations of *R. korthalsii* extract and the T and NK cell population in the control and extract treated PBMC were identified by immunophenotyping. Intracellular perforin and granzyme B expressions were detected by flow cytometry and extra-cellular Granzyme B, IFN-γ and TNF-α production in the isolated NK cells were determined by ELISA. The cytotoxicity of effector NK cell towards target K562 cell was assessed by CytoTox 96 assay.

**Results:**

*Rhaphidophora korthalsii* methanol extract significantly increased PBMC NK cell population and intracellular perforin and granzyme B expressions. Moreover, the extract also enhanced the secretion of IFN-γ and TNF-α which subsequently enhanced the cytotoxicity of NK cell against the NK sensitive target K562 cell line. NK cell enriched with extract treated PBMC showed better activation than NK cell directly treated with the extract.

**Conclusion:**

Our findings indicated a potential IL-2 free immunotherapy through direct and indirect *R. korthalsii* stimulation on NK cell activation.

## Background

Immunostimulator is one type of immunomodulator which can strengthen the body’s immune system [1]. One of the research directions in complementary medicine is to discover a safer and effective immunostimulators which can help in preventing diseases and to be used in clinical practice for patients with immune disorders. Recent years, the focus on the study of biological activities such as immunostimulatory effect from plant extracts based on ethnopharmacology has been revived all over the world due to the low or absence of toxicity, complete biodegradability, availability from renewable resources and low production cost in comparison to pharmaceutical compounds [2-4].

*Rhaphidophora korthalsii,* Schott. is a traditional herb under the family of Araceae. It can be widely found throughout Asia region such as India, Sri Lanka, Cambodia, Venezuela, Malaysia, Australia and Indonesia [5]. *R. korthalsii* has been traditionally used for cancers and skin diseases treatment. Earlier studies have found that *R. korthalsii* extract possesses selective cytotoxic and antioxidant effects. For example, the extract was found to suppress P388, Molt 4, KB, SW 620 and T-47D cell lines. The antioxidant activity of the extract had subsequently contributed to the enrichment of macrophage cell number [6-8]. On top of that, the extract had also showed to exert immunomodulatory effect on mice splenocyte and human peripheral blood mononuclear cell (PBMC) proliferation, increase of the NK cell population, cytokines secretion and cytotoxicity *in vitro* and *in vivo*[8-11].

NK cells are believed to play an important role as effector cells to lyse cancerous target when compared to other types of lymphocytes in human immune system. This is because NK cell forms an early immune defense during innate immunity before the emergence of T and B lymphocytes upon the entrance of pathogen or formation of tumor [12]. To date, the majority of the studies reported on immunoregulation of *R. korthalsii* were using a mix population of lymphocytes. However, little or no information is available on the modulation of *R. korthalsii* against targeted lymphocyte population especially the effects on NK cell. To address this question, this study was carried out to present the immunoregulatory effect of *R. korthalsii* on human PBMC NK cell activation and cytotoxicity.

## Methods

### Reagents and chemicals

Fluoroisothiocyanate (FITC)- labeled CD16, CD3, perforin, granzyme B, and phycoerytherin (PE)-labeled CD56 and IL-2 neutralizing MQ1-17H12 monoclonal antibodies, GolgiStop™ solution and Perm/Wash™ solution were purchased from BD, USA; isotype control mouse IgG1 and FITC goat anti-rabbit Ig were purchased from Abcam, USA; Enzyme Link Immunosorbent Assay (ELISA) kit for IFN-γ, TNF-α and Granzyme B cytokines from Bender MedSystems, Austria; NK cell and T cell isolation kits from Milteny Biotech, Germany; human recombinant human Interleukin 2 (rhIL-2), trypan blue solution, DMEM were brought from Sigma, USA.

#### Preparation of the methanol extract of *R. korthalsii*

*R. korthalsii* methanol extract was prepared using the same extraction method as stated in our earlier study [9]. *R. korthalsii* leaves (voucher no: FRIM 33687) were collected from Georgetown Herbal Farm, Penang, Malaysia and was authenticated by Mr. Lim Chung Lu from the Forestry Division of the Forest Research Institute of Malaysia (Kepong, Selangor). The leaves were then air-dried and extracted using 250 mL of methanol (J.T. Baker, USA) for 72 hours. The extract was filtered with Whatman filter paper no 1 and evaporated to dryness under reduced pressure using Aspirator A-3S (EYELA, Japan) at < 40°C. This process was repeated three times (yield 27.3%, w/w) respectively. The methanol extract was dissolved in DMSO (Sigma, USA) at a concentration of 10 mg/mL.

### Cell line and cell preparation

PBMC was isolated from five different blood donors using Ficoll-Plaque Plus (Amersham Biosciences, USA) according to the manufacturer’s protocol. Informed consent was acquired from all donors and this work was carried out in compliance with the Helsinki Declaration for ethical approaches of conducting scientific research. This study was approved by the ethical committee of University Putra Malaysia. In brief, one ratio of diluted blood (1:1 ratio in PBS) sample was layered on 2 ratio of Ficoll-Plaque Plus (Amersham Biosciences, USA), pelleted (400 *g*, 40 minutes at 18-20°C) and the buffy coat was collected, washed twice with PBS and cultured in DMEM (Sigma, USA) (100 IU/mL of penicillin, 100 μg/mL of streptomycin [Flowlab, Australia] and 10% v/v Fetal Bovine Serum [FBS] [PAA, Austria]). PBMC with viability higher than 90% was subjected to the following assays.

### T cell and NK cell immunophenotyping for human PBMC

The T cell and NK population in the control and extract treated PBMC were identified using flow cytometry surface antigen immunophenotyping. In brief, PBMC (5 × 10^5^ cells/mL) was exposed to various concentrations of *R. korthalsii* methanol extract (50 μg/mL, 25 μg/mL or 1 μg/mL) for 24, 48 and 72 hours. Con-A (1 μg/mL) and LPS (1 μg/mL) treated PBMC and untreated PBMC were used as positive and negative controls respectively. All samples were then washed, double stained with 10 μg/10 μL of conjugated anti-CD3 FITC (isotype control: Mouse IgG2a κ) and 12.5 μg/10 μL of conjugated anti-CD56 PE antibodies (isotype control: Mouse IgG1 κ) and the population of CD3^+^ T cell and CD56^+^ NK cell were analyzed using BD FACSCalibur™ flow cytometer with BD CellQuest™ software (BD Biosciences, USA).

### NK cell Granzyme B and perforin expressions

PBMC was treated with *R. korthalsii* extract at 25 μg/mL or rhIL-2 at 200 U/mL for 72 hours in 6 well plate (BD Biosciences, USA). 2 hours before the end of the incubation period, the production of perforin and granzyme B were inhibited by adding and mixing with 2 μL of BD GolgiStop™ into each experimental well. Then, 1 × 10^6^ cells were harvested, washed with 1 mL of PBS and stained with 12.5 μg/10 μL of conjugated anti-CD 56-PE antibody in a final volume of 100 μL PBS for 30 minutes at 4°C. After that, the cells were washed and incubated with 250 μL of Fixation/Permeabilization solution (BD Biosciences, USA) for 20 minutes at 4°C. Subsequently, the cells were then washed twice with BD Perm/Wash™ buffer, incubated with 5 μL of either 10 μg/10 μL of mouse anti-human Granzyme B-FITC (Isotype control: Mouse IgG1, κ) (BD Biosciences, USA) or 10 μg/10 μL of mouse anti-human Perforin-FITC antibody (Isotype control: Mouse IgG2b, κ) (BD Biosciences, USA), stained with second antibodies and analyzed using BD FACSCalibur™ flow cytometer with BD CellQuest™ software (BD Biosciences, USA).

### NK cell Isolation

**Figure 1:**
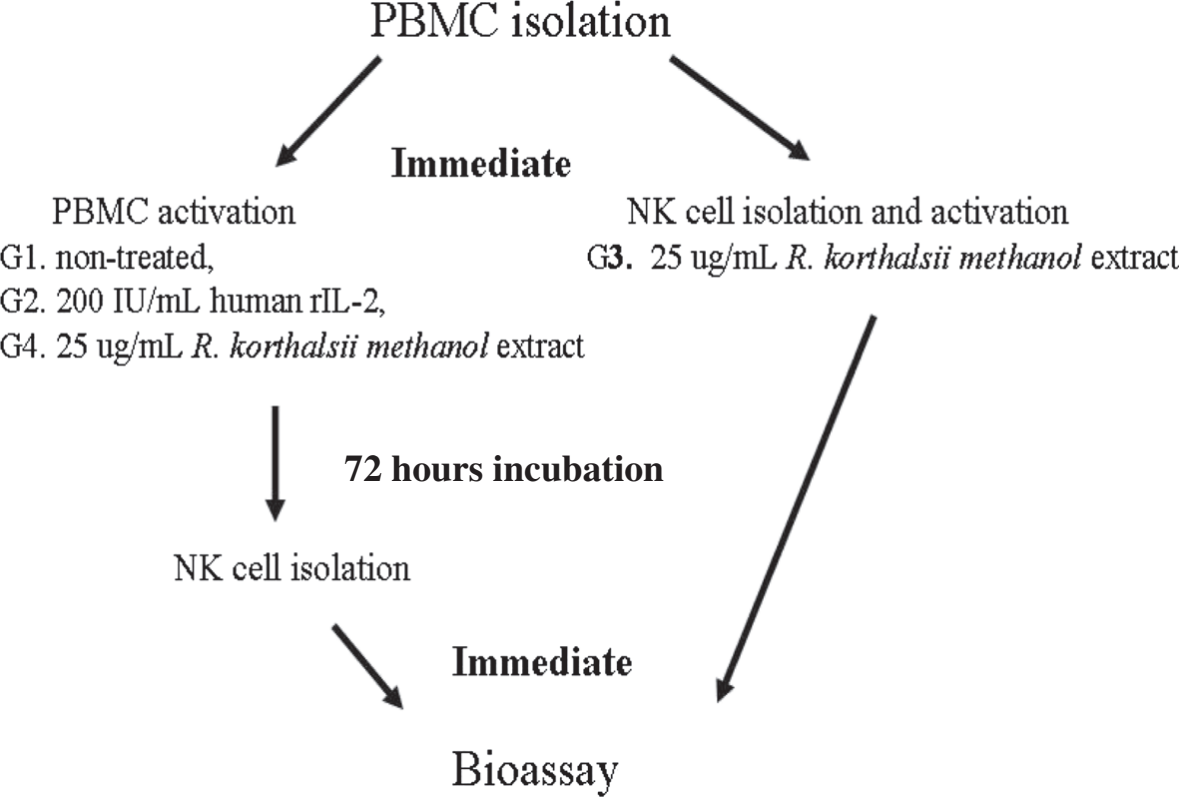
**Brief description of NK cells treatment.** G1 to G4 are the groups of treatment. Direct and indirect activations of *R. korthalsii* against NK cells were studied through isolation of NK cells before and after 72-hour incubation with the extract.

The PBMC was subjected to activation and treatment as stated below and summarised in Figure 1.

Group 1: non-treated PBMC was cultured for 72 hours and subjected to NK cell magnetic isolation

Group 2: PBMC was treated with 200 IU/mL of human rIL-2 treated for 72 hours before subjected to NK cell isolation

Group 3: Direct NK cells activation whereby freshly isolated PBMC was subjected to NK cell isolation and was further cultured in 25 μg/mL of *R. korthalsii* methanol extract for 72 hours.

Group 4: Indirect NK cell activation whereby PBMC was treated with 25 μg/mL of *R. korthalsii* methanol extract for 72 hours before subjected to NK cell magnetic isolation.

NK cell isolation kit by immunomagnetic negative selection (Milteny Biotech, Germany) was used to isolate primary NK cells from isolated PBMC. In brief, either the freshly isolated PBMC (for direct *R. korthalsii* methanol extract treatment) or the PBMC cultured 3 days with different treatment was magnetically labeled with a cocktail of NK cell Biotin-Antibody Cocktail followed by NK cell MicroBead Cocktail. The magnetically labeled cells were then separated using MS column in the magnetic field of the MACS separator. Purity of NK cells was quantified by using flow cytometry where the entire isolated NK cell was labeled with anti-CD16-FITC (isotype control: Mouse IgG1 κ), anti-CD-56-PE (isotype control: Mouse IgG1 κ) and analyzed by flow cytometer (BD Biosciences, USA). Isolated NK cell with purity more than 95% were used for further studies.

### ELISA for determination of extra-cellular Granzyme B, IFN-γ and TNF-α

Expressions of Granzyme B, IFN-γ and TNF-α were performed using Cytokine Enzyme Link Immunosorbent Assay (ELISA) kit (Bender MedSystems, Austria). In brief, the extracted human NK cells after 72 hours of different treatments were cultured for another 24 hours without treatment. Another group of direct and indirect extract treated NK cells were treated with 15 μg/mL of MQ1-17H12 antibody (BD, USA) to neutralize the activity of IL-2 contributed mainly by the surrounding cell especially in the indirect extract treated NK cell. Similarly, CD3 T cells were isolated using CD3 T cell isolation kit by immunomagnetic positive selection based on the same grouping for the NK cell in Figure 1. The supernatant of these different control and treatment groups of T cell were subjected to ELISA determination of extra-cellular Granzyme B, IFN-γ and TNF-α levels and compared their effects with the treatments on the NK cell. The supernatant from each group was collected, added into the pre-coated plate of the kit and incubated for 3 hours. After that, these samples were washed and immediately added with 3,3′,5,5″-tetramethylbenzidine (TMB) Peroxidase substrate followed by 1 M Phosphoric acid stop solution. The ELISA plate was read at absorbance OD of 450 and 620 nm wavelengths using a μQuant ELISA Reader (Bio-Tek Instruments, USA). The results were expressed in pg/mL. Each experimental and control sample was assayed in three biological replicates. Data were expressed as pg/mL.

### *In vitro* cytotoxicity of NK cell on NK cell sensitive K562 cell line

The cytotoxicity of effector NK cell towards target K562 cell in the ratio of 2:1 and 10:1 for 24 hours was determined using CytoTox 96 nonradioactive cytotoxicity assay kit (Promega, USA) according to the manufacturer’s protocol. The percentage of cytotoxicity was calculated using the formula below

$$\%\;\mathrm{Cytotoxicity}=\frac{\mathrm{OD}\;\mathrm{sample}-\mathrm{OD}\;\mathrm{target}\;\mathrm{spontaneous}-\mathrm{OD}\;\mathrm{effector}\;\mathrm{spontaneous}}{\mathrm{OD}\;\mathrm{target}\;\mathrm{spontaneous}-\mathrm{OD}\;\mathrm{effector}\;\mathrm{spontaneous}}\times 100$$

### Statistical analysis

All tests were carried out with 3 independent experiments and each of the experiment consisted of 3 technical replicates. All results are expressed as Mean ± Standard Error (S.E.M.). Significant levels (p < 0.05) were evaluated using ANOVA test (one way) followed by post hoc Duncan test.

## Results

### *R. korthalsii* methanol extract increased the PBMC NK cell population

The changes of CD3^+^ T cell and CD56^+^ NK cell composition in PBMC after treated with *R. korthalsii* methanol extract were analyzed using flow cytometry. Based on the results in Table 1, human PBMC incubated with 25 μg/mL of extract led to a decreased number of the T cell population (CD3^+^CD56^-^) by 1.13 times but significantly (*P* < 0.05) increased the NK cell (CD3^-^CD56^+^) population by 1.5 times. On the other hand, 50 μg/mL of extract stimulated the NK cell population while maintaining the T cell population which contributed to less significant changes of the NK cell (CD3^-^CD56^+^) population as compared to 25 μg/mL of extract. Thus, 25 μg/mL of extract stimulated proliferation of NK cell rather than T cell in the PBMC.

**Table 1:** **T cell and NK cell immunophenotyping on human PBMC after treating with various concentrations of *R. korthalsii *methanol extract or positive controls for 72 hours**

	**T cell or NK cell population (%)**	
	**CD 3**^**+ **^**CD56**^**-**^	**CD3**^**- **^**CD56**^**+**^
Control	55.8 ± 1.3^x^	9.6 ± 2.7^a^
*R. korthalsii* (50 μg/mL)	52.1 ± 2.7 ^x^	12.1 ± 1.3^a,b^
*R. korthalsii* (25 μg/mL)	49.0 ± 1.6^y^	17.5 ± 3.^b^
*R. korthalsii* (1 μg/mL)	54.3 ± 1.9^x^	8.9 ± 1.2^a^
rhIL-2 (200 IU/mL)	56.2 ± 2.5^x^	14.1 ± 1.7^b^
Con-A (1 μg/ml)	61.0 ± 2.4^z^	6.7 ± 1.4^c^
LPS (1 μg/ml)	47.9 ± 1.1^y^	7.8 ± 1.9^a,c^

Values (n = 9; 3 independent experiments with three technical replicates each) represent the means ± S.E.M. x, y and z indicate significant different among the groups for CD 3^+^CD56^-^ (T lymphocyte) while a, b and c indicate significant different among the groups for CD 3^-^CD56^+^ (NK cell) (P < 0.05).

### *R. korthalsii* methanol extract enhanced NK cell perforin and granzyme B expressions

**Figure 2:**
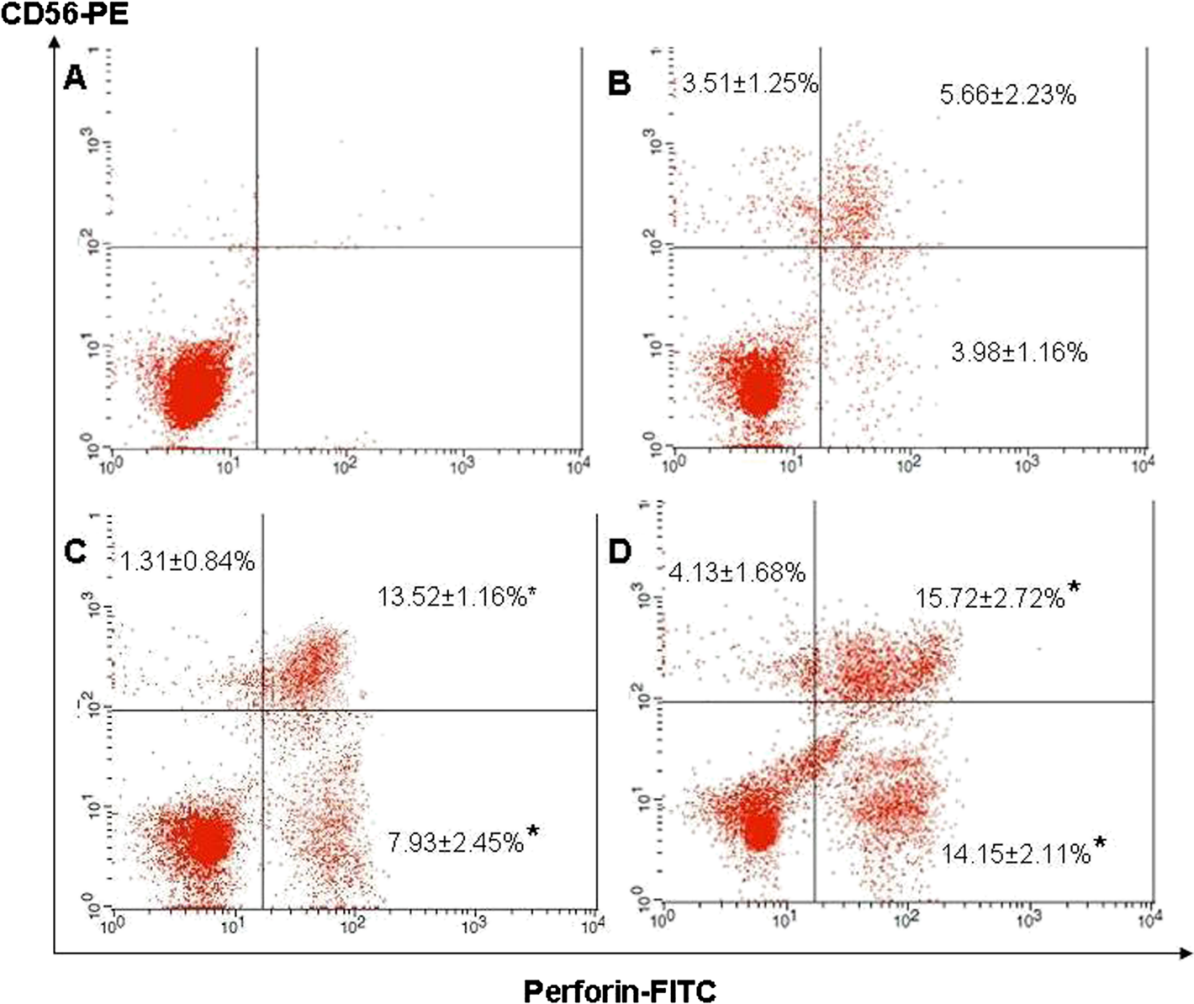
**CD 56 and Perforin expression of (A) isotype control (B) untreated, (C) rhIL-2 treated or (D) *R. korthalsii *methanol extract treated PBMC.**Values (n = 9; 3 independent experiments with 3 technical replicates each) represent the means ± S.E.M. * Significant (P < 0.05).

**Figure 3:**
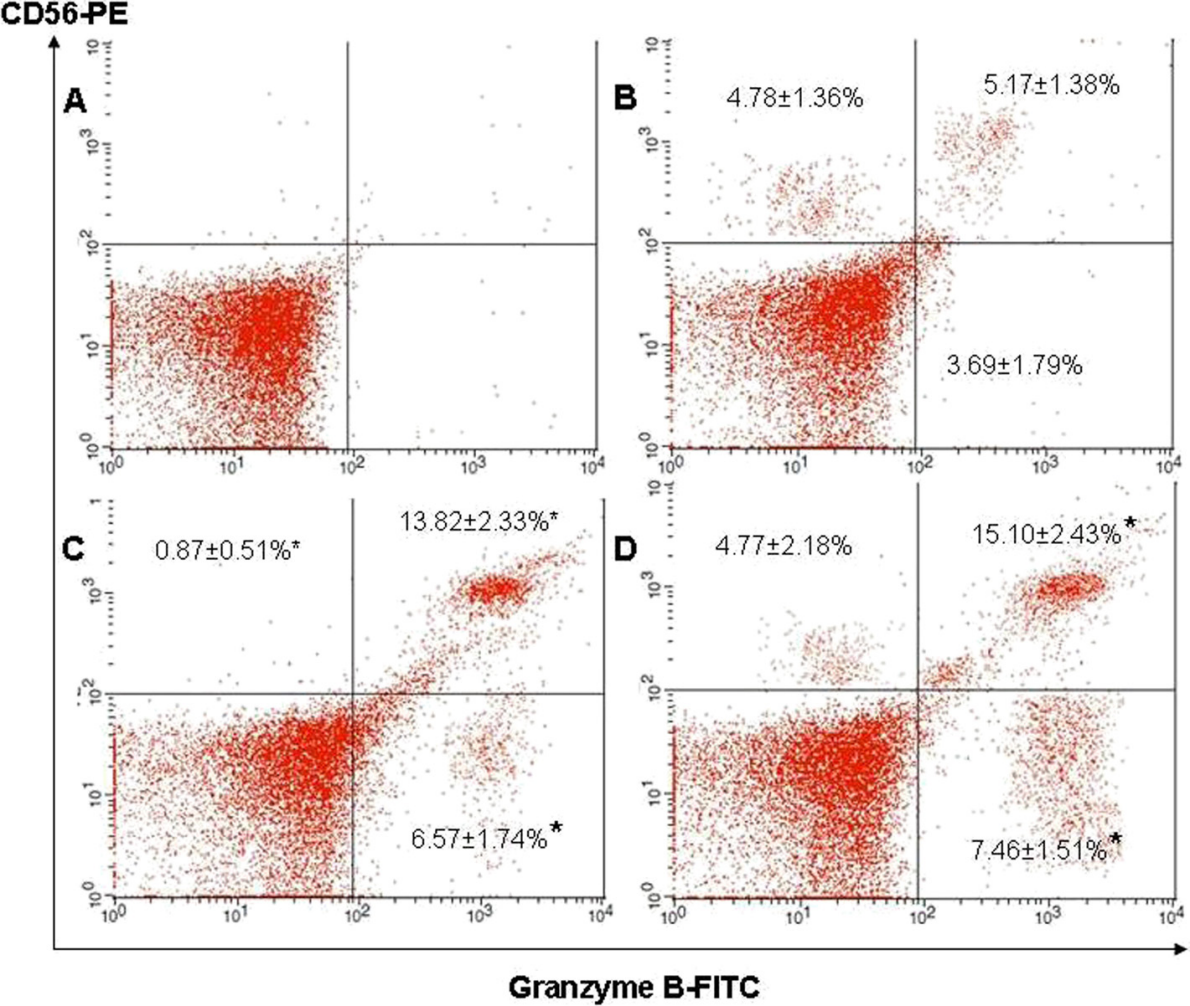
**CD 56 and Granzyme B expression of (A) isotype control (B) untreated, (C) rhIL-2 treated or (D) *R. korthalsii *methanol extract treated PBMC.**Values (n = 9; 3 independent experiments with 3 technical replicates each) represent the means ± S.E.M. * Significant (P < 0.05).

Since *R. korthalsii* methanol extract activated PBMC showed significantly higher level of NK cell numbers, NK cell endocytosis production of the apoptosis or necrosis inducing proteins (Granzyme B and Perforin) in PBMC were quantified to explore the contribution of NK cells in PBMC cytotoxicity. As shown in Figure 2 and Figure 3, the rIL-2 and extract treated PBMC showed a significantly higher level of perforin or granzyme B co-expressing CD56^+^ cells (13.52 ± 1.16% and 13.82 ± 2.33% for rIL-2 treated PBMC; 15.72 ± 2.72% and 15.10 ± 2.73% for extract treated PBMC). The frequencies of perforin^+^/CD 56^-^ and Granzyme B^+^/CD 56^-^ populations were only upregulated by the extract treated PBMC but not significant by rhIL-2 treated PBMC (Figures 2 and 3). From these results, both rIL-2 and extract were able to stimulate higher percentage of NK cell population with majority of CD56^+^ (> 75 %) co-expressed perforin and granzyme B as compared to control (~61% for perforin and ~51% for granzyme B) (This was calculated based on the CD56^+^ Perforin or Granzyme B^+^ population divided by Total CD56^+^ population from Figures 2 and 3).

### *R. korthalsii* methanol extract enhanced CD56^bright^CD16^dim^ NK cell population

**Figure 4:**
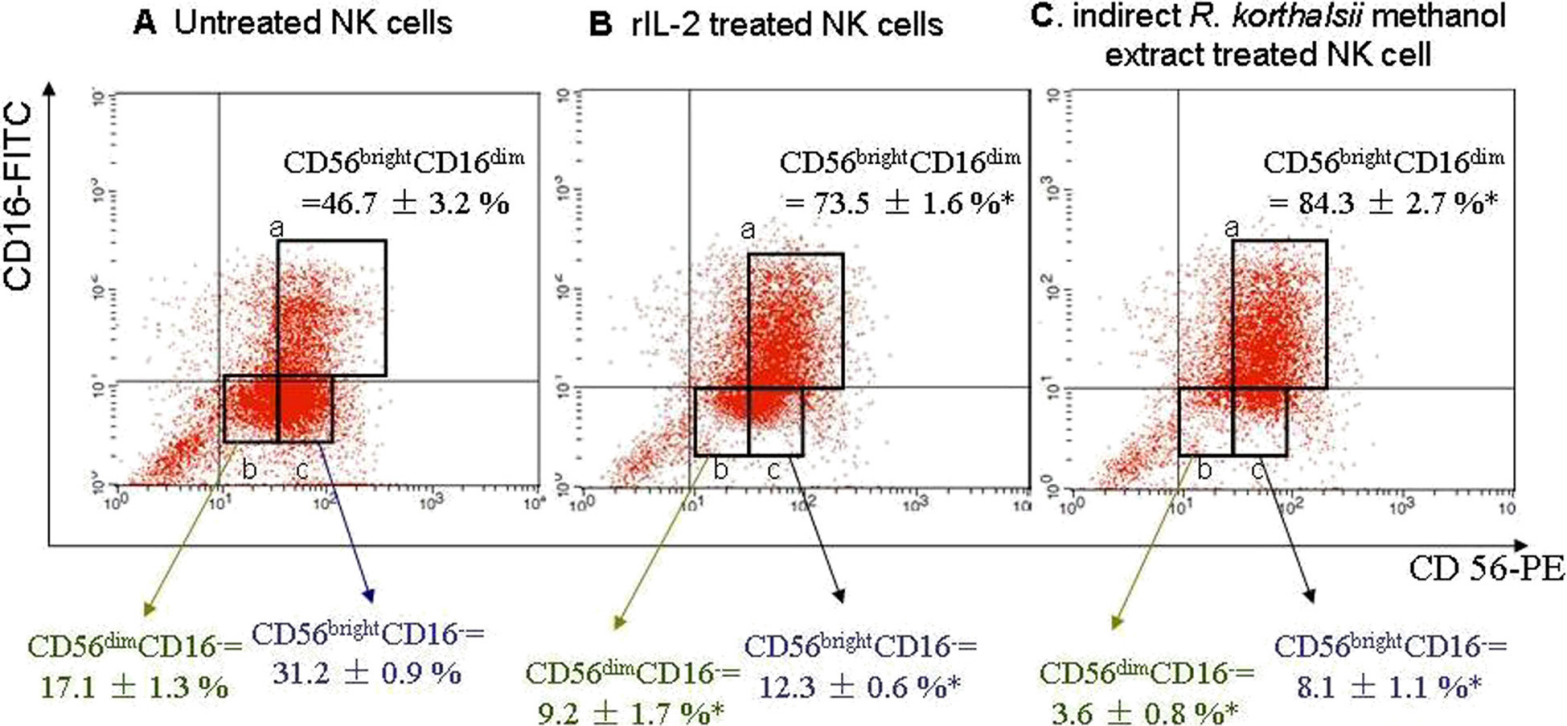
**Flow cytometry analysis of CD56 and CD16 expression of NK cells isolated from treated and non-treated PBMC by using immunomagnetic negative selection NK cell isolation kit (Milteny Biotech, Germany).** Gradual increased of NK cells co-expressed the CD56^bright^ and CD16^dim^ were observed in A to C. **A**. Untreated NK cells, **B**. rIL-2 treated NK cells, **C**. indirect *R. korthalsii* methanol extract treated NK cell. Values (n = 9; 3 independent experiments with 3 technical replicates each) represent the means ± S.E.M. * Significant (P < 0.05).

At least 95% of the isolated cells was CD56^+^ as shown in the upper and lower right columns of Figure 4. Treatments with rIL-2 and *R. korthalsii* extract were able to increase CD56^bright^CD16^dim^ NK cell population.

### *R. korthalsii* favored NK cell IFN-γ and TNF-α secretion

Together with the induction of intracellular perforin and granzyme B expressions, *R. korthalsii* methanol extract was also able to stimulate extracellular IFN-γ and TNF-α production either by direct or indirect stimulation of the NK cells. When NK cells were cultured with 72 hours of extract, the expression of IFN-γ and TNF-α were increased markedly as compared to the untreated control group or even with the human rIL-2 treated group whereas the secretion of granzyme B was not affected (Table 2). The direct extract activated NK cells showed a lower level of IFN-γ and TNF-α production as compared to indirect activated NK cells. This might be contributed by the uptake of cytokine (e.g. IL-2) produced by the neighbouring lymphocytes which were also stimulated by the extract in the same time. Thus, the level of IFN-γ and TNF-α production were found to be the highest in the indirect extract treated NK cells followed by the direct extract treated NK cells and human rIL-2 treated NK cells. These results were supported by the reduction of IFN-γ and TNF-α secretions in the indirect extract treated NK cells to comparable level as the direct extract treated NK cells with the presence of IL-2 neutralizing antibody MQ1-17H12 (BD, USA).

**Table 2:** **ELISA Granzyme B, TNF-α and IFN-γ detection of untreated, human rIL-2 treated, direct or indirect *R. korthalsii *methanol extract stimulated Human NK cells**

	**Granzyme B**	**TNF-α**	**IFN-γ**
	**(pg/mL)**		
CD56 NK cell			
Untreated NK cells	72.3 ± 9.5^i^	89.1 ± 11.3^x^	857.5 ± 19.5^a^
Human rIL-2 treated NK cells	86.2 ± 4.1^i^	743.3 ± 15.6^y^	3672.6 ± 28.1^b^
Direct *R. korthalsii* extract treated NK cells	79.1 ± 8.5^i^	478.9 ± 17.8^z^	2954.2 ± 27.9^c^
Indirect *R. korthalsii* extract treated NK cells	88.5 ± 7.3^i^	814.1 ± 21.3^zz^	6238.8 ± 38.2^d^
Direct *R. korthalsii* extract treated NK cells + MQ1-17H12 antibody	78.4 ± 5.7^i^	442.9 ± 26.5^z^	2713.9 ± 33.5^c^
Indirect *R. korthalsii* extract treated NK cells + MQ1-17H12 antibody	83.6 ± 6.2^i^	527.4 ± 31.3^z^	3112.2 ± 41.7^c^
CD3 T cell			
Untreated CD3 T cells	66.4 ± 4.7^i^	84.5 ± 9.3 ^x^	691.2 ± 28.5^e^
Human rIL-2 treated CD3 T cells	69.8 ± 5.2^i^	876 ± 24.7^zz^	2976 ± 36.1^c^
Direct *R. korthalsii* extract treated CD3 T cells	65.3 ± 7.1^i^	215.8 ± 41.2^zzz^	977.2 ± 28.4^f^
Indirect *R. korthalsii* extract treated CD3 T cells	68.2 ± 8.3^i^	164.5 ± 33.9^zzz^	756.3 ± 23.9 ^e^

Values (n = 9; 3 independent experiments with three technical replicates each) represent the means ± S.E.M. Significant (P < 0.05). i indicate no significant different among the groups for Granzyme B; x, y, z, zz, zzz indicate significant different among the groups for TNF-α while a, b, c. d. e and f indicate significant different among the groups for IFN-γ (P < 0.05).

### *R. korthalsii* enhanced NK cell cytotoxicity

**Figure 5:**
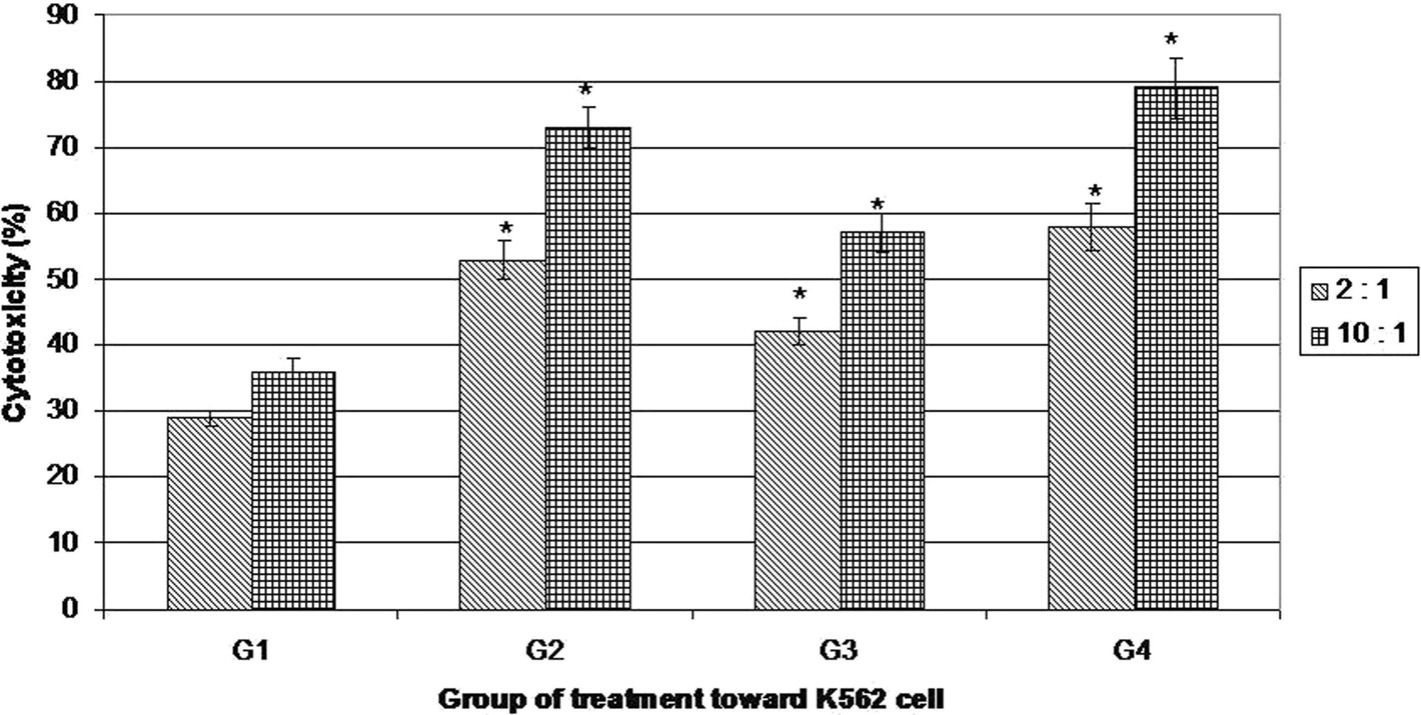
**Cytotoxicity of treated or non-treated NK cells towards K562 cell at ratio of effector NK cell to target K562 cell at 2:1 and 10:1 evaluated by LDH microcytotoxicity assay.** G1 is untreated NK cells; G2 is Human rIL-2 treated NK cells; G3 is direct *R. korthalsii* extract treated NK cells; G4 is indirect *R. korthalsii* extract treated NK cells. Each value represents the means ± S.E.M. for 3 independent experiments with 3 technical replicates each. The differences between the control group and treated group were determined by one-way ANOVA (*P < 0.05).

The exposure of human natural killer cell to *R. korthalsii* extract directly or indirectly were associated with the activation of NK cell activity against the targeted cancer cell. A standard LDH microcytotoxicity assay was subsequently performed to quantify the cytotoxicity of the treated and non-treated NK cells against NK cell sensitive K562 cell line. Figure 5 showed that LDH was released in the culture medium following a 24 hours of treated or non-treated NK cell exposures towards K562 cell at the ratio of 2:1 and 10:1. Two-hour-exposure of K562 cells with NK cells treated directly or indirectly with 25 μg/mL of *R. korthalsii* methanol extract raised LDH leakage to 42 ± 1.7% and 58 ± 4.1% respectively. The cytotoxicity was further increased when the number of NK cells increased from 2 times to 10 times against the total cell number of K562. All of the treated NK cells showed better cytotoxicity against K562 cells as compared to untreated NK cells with the differential sensitivities of the treatments in the following order: *R. korthalsii* indirect activated NK cells > rhIL-2 indirect treated NK cells > *R. korthalsii* direct activated NK cells > untreated NK cells.

## Discussion

An immunomodulator is a compound or substance that is capable of either up-regulating or down-regulating a specific aspect of the host immune system. IL-2 has been identified as the NK cell immunomodulator in tumor immunotherapy. However, low concentration of IL-2 was failed to induce adequate amount of immune cell cytotoxicity towards tumor regression while high concentration of IL-2 can produce a severe lymophopenia and toxic side effect [13,14]. Thus, searching for novel immunomodulator which can work synergistically with IL-2 to reduce or replace its working dosage has been proposed from time to time and this may contribute to the success of adoptive immunotherapy in cancer treatment. Previously, the *in vitro* and *in vivo* proliferation and cytokine secretion studies have showed that *R. korthalsii* methanol extract has no suppressive effect but on the other hand has stimulation effect on mice and human lymphocyte function [8-11]. In this study, *R. korthalsii* methanol extract had stimulated NK cell population in a dosage dependent manner (Table 1). Low concentration of extract (1 μg/mL) did not alter significant changes (p < 0.05) on both T and NK cell population. However, 25 μg/mL specifically promoted the increased of NK cell population which associated with slight reduction of T cell population. High concentration of extract possessed similar effect as rIL-2 where the increased of NK cell population was associated with the maintenance of high population of T cell. This phenomenon has suggested that high concentration of *R. korthalsii* methanol extract can work similarly as rIL-2 to stimulate both population of T and NK cell [15] while 25 μg/mL of extract selectively stimulated NK cell more than T cell.

In this study, our results showed that extract enhanced the production of perforin and granzyme B on both CD56^+^ and CD56^-^ populations in PBMC (Figures 2 and 3). Thus, up-regulation of PBMC cytotoxicity against tumor target hepatoblastoma HepG2 cell and erythroleukemia K562 cell line [9-11] may be contributed by either the NK (CD56^+^) or non-NK (cytolytic T which is CD56^-^) killer cells. Previously, Wahlberg et al. [16] showed that NK cells were the sole effector cells actively involved in killing the tumor cell target as compared to other potential cytotoxic immune effector cells such as Tumor-specific cytotoxic T lymphocytes and cytotoxic NK-T cells. K562 cells are tumor target cells which are sensitive to NK cell cytolytic activity. Thus, the reduction of the K562 cells’ viability might be contributed by the NK cell cytolytic activity which was further upregulated by the *R. korthalsii* methanol extract. NK cells are large granular lymphocytes in the innate immunity which kill the tumor or virus infected target cells by using three mechanisms [17]. The first mechanism is by secreting granules which contain perforin and granzymes that induce necrosis and apoptosis respectively. Perforin is a killer-cell specific cytolytic mediator produced by cytotoxic lymphocytes and stored in cytoplasmic granules. Both perforin and granzyme are originated from cytolytic serine-proteinases family and work synergistically to lyse the cell targeted by cytolytic lymphocytes such as cytolytic T and NK cells. These proteins were reported previously to be present in the rIL-2 induced PBMC and were degranulated towards the target cells during cell mediated cytotoxicity [18]. IL-2 was previously found to be secreted upon extract activation [11] at a lower concentration as compared to the rIL-2 which was used as the control in this study. Thus, it is possible that up-regulation of perforin and granzyme B production in the NK cells maybe stimulated via both extract or IL-2 produced by extract stimulated lymphocytes. The second mechanism is by TNF receptor family ligand interaction such as Fas ligand (Fas L)/Fas pathway [19] and the third would be the secretion of antitumor cytokine such as IFN-γ. Previous study showed that HepG2 was resistant towards the TNF family Fas L/Fas induced apoptosis pathway of NK cell but sensitive to NK cell granule dependent necrotic death [20]. It was found that 72 hours of extract activation also induced high production of extra-cellular T helper 1 cytokine, IFN-γ [11]. The question to this phenomenon was which population of cells in PBMC was contributed to the up-regulation of IFN-γ secretion upon extract stimulation. The immunophenotyping of PBMC postulated that high expression of IFN-γ may be contributed by NK cell population rather than T cell. This was because at that specific concentration, *R. korthalsii* methanol extract had up-regulated the NK cell population while reduced the T cell population in the CD3 and CD56 immunophenotyping of the extract treated or none treated PBMC (Table 1). Modulation on IFN-γ production has previously reported to affect NK cell cytotoxicity [21]. Thus, enhancement of immune cell cytolytic activity may be contributed by the synergistic effect of both cytolytic proteins (granzyme B and perforin) and also antitumor cytokine (IFN-γ). Although the exact mechanism of *R. korthalsii* methanol extract in activating immune cell cytotoxicity is yet to be fully understood, it is expected that the activation may be due to the direct binding effect of the extract to NK cells or indirect effect of IL-2 secreted by accessory cells in PBMC. To confirm the activation of extract on NK cell, CD56 enriched NK cell population was isolated from PBMC and subjected to investigation of perforin, granzyme B and cytokine expressions.

Different treatment groups were setup in order to elucidate the mechanisms of cytotoxicity activity and NK cell activation by *R. korthlasii* methanol extract. We have previously showed that extract-treated PBMC secreted higher level of IL-2 production as compared to the non-treated cells. This result gave an insight that the extract may not only activate NK cells but also other types of immune cells in the PBMC culture. Moreover, CD4 T cell and NK cells were found to work synergistically towards tumor removal [22]. Thus, in this study, the treated and non-treated PBMC were cultured for 72 hours prior to NK cell isolation to allow cell-to-cell interactions for better cytotoxic effect towards tumor cells. On the other hand, NK cells which express CD56^+^CD16^−/+^ were also isolated from fresh PBMC (Figure 1) before treated with *R. korthalsii* methanol extract for a better understanding of the immunoregulatory effect contributed by direct interaction between the extract and NK cells. NK cells directly isolated from fresh blood for direct extract activation shared similar 1:1 ratio of CD16^+^ and CD16^-^ population as in the non-treated PBMC isolated NK cells (Figure 4). As compared to untreated PBMC, NK cells isolated from the rIL-2 or extract treated PBMC showed an increment of CD56^bright^CD16^dim^ population. CD56^bright^CD16^dim^ NK cell population plays an important role to produce cytokines such as IFN-γ and only possess weak cytotoxicity against tumor [23]. The increment of this population (Figure 4) after stimulated by rIL-2 and *R. korthalsii* methanol extract had strong correlation with higher production (Table 2).

Our previous studies have demonstrated that *R. korthalsii* methanol extract could enhance immune cells proliferation, Th1 cytokine expression and cytotoxicity. In this study, apart from the direct activation of the *R. korthalsii* methanol extract on the NK cells isolated from fresh human blood, the effect of the extract on NK cells was also monitored through isolation of NK cells from the pre-activated PBMC. This approach of activation was chosen because immune cells activation involves a signaling cascade from innate immunity to adaptive immunity which includes multiple types of immune cells. For example, a change in dendritic cells self-markers can trigger NK cells activation which further promotes the activation of dendritic cells and lead to the priming of Th1 responses [24]. From the results obtained, *R. korthalsii* methanol extract had demonstrated both direct and indirect immunoregulatory effect on human NK cell cytotoxicity *in vitro*.

Although CD56^bright^CD16^dim^ NK cell population possessed less cytotoxicity against tumor as compared to CD56^dim^ NK cell, the present of cytokines especially IL-2 can significantly improved the cytotoxicity of all subtypes of NK cell [23]. Thus, isolated NK cell treated with rIL-2 and extract were observed to have higher cytotoxicity against K562 cell. Besides, the way NK cells counter react with tumor involved not only the NK cells’ cytolytic activity but also those of the other cytokines. Krishnaraj and Bhooma [25] found that senescent NK cells showed cytokine secretory deficiency which contributed to tumor establishment and viral infection although NK cell cytotoxicity remained the same. In this study, the enhancement of IFN-γ and TNF-α production by the extract activated NK cells was confirmed by ELISA. The results showed that the level of IFN-γ expression by pure NK cell population from either extract direct or indirect activation were quite similar to the level of IFN-γ expressed by extract activated PBMC in our previous report [11]. Furthermore, IFN-γ and TNF-α productions in extract treated isolated CD3 T cell were much lower as compared to NK cell. This result gave the idea that IFN-γ produced by extract activated PBMC was contributed mainly by the extract direct or indirect activated NK cells and this results was supported by the IL-2 neutralizing groups where addition of MQ1-17H12 antibody only affected the secretion of IFN-γ and TNF-α for the indirect extract treated NK cell. IFN-γ is a multifunctional cytokine with wide stimulatory effects on anti-tumoral immune reactions either through direct cytotoxic effect on tumor cells or indirect stimulation of immune effector cells such as macrophages and NK cells to target on the tumor. IFN-γ mediates NK cells’ cytolytic activity through the increase of the ULBP level of the tumors cells which then binds to the NKG2D [26]. Binding of RAET1G3 which is the variant of ULBPs expressed by HepG2 was found to stimulate NK cells to further enhance IFN-γ secretion [27]. This idea had raised the possibility that IFN-γ production stimulated by *R. korthasii* methanol extract might contribute to the activation of NK cells’ cytotoxicity against HepG2 and K562 [9-11]. Enhancement of the NK cell cytolytic activity toward K562 cell by the extract was confirmed in this study. However, IFN-γ by itself did not demonstrate a sufficient amount of cytotoxicity to kill the tumor cells. Aid from other cytokines such as TNF-α could have overcome this barrier [28]. Thus, IFN-γ, TNF-α, granzyme B and perforin stimulation may contribute to a higher level of NK cells’ cytotoxicity towards K562 cells as compared to the untreated NK cells. All of the above results showed strong evidence that activation of *R. korthalsii* methanol extract contributes to the enhancement of NK cell cytotoxicity. In this study, no significant amount of granzyme B was detected in the supernatant of the culture. This result carried the message that spontaneous leakage of granzyme B did not take place in all of the untreated and treated NK cells. Leakage of Granzyme B into cytosol and extracellular environment was found to induce NK cell self-apoptosis, which is termed “Granzyme B leakage-induced cell death” (GLCD) [29]. Thus, GLCD did not occur in all of the untreated or treated NK cells in this experiment as no significant leakage of Granzyme B was detected.

## Conclusion

Our study gave strong indication that synergistic effects of *R. korthalsii* methanol extract and IL-2 expressed by the neighouring cells in the PBMC population produced a greater net activation over NK cells. NK cells isolated from the PBMC treated with *R. korthalsii* methanol extract resulted in a higher expressions of perforin, granzyme B, IFN-γ, TNF-α, in comparison to those that were treated with rIL-2 alone or PBMC isolated NK cell directly treated with the extract. This finding suggested the potential future use of *R. korthalsii* methanol extract as an alternative to mono-immunotherapy by IL-2 in adoptive immunotherapy. Future studies should focus on the isolation of the active compounds present in *R. korthalsii* and the evaluation of the effects of these compounds on activation and inhibition markers of NK cell.

## Competing interests

The authors declare that they have no competing interests.

## Authors’ contributions

SKY conceived the study, carried out the experimentation, acquisition and analysis of data and drafting of the manuscript. WYH and BKB assisted with the concept, analysis of data and drafting of the manuscript. AMA and NBA provided funding. ARO, AMA and NBA conceived, designed and supervised the study and revised the manuscript. All authors have read and approved the final manuscript.

## Pre-publication history

The pre-publication history for this paper can be accessed here:

http://www.biomedcentral.com/1472-6882/13/145/prepub
